# Dermoscopy and the Early Melanoma Concept

**DOI:** 10.1111/ijd.70546

**Published:** 2026-09-29

**Authors:** Camila Scharf, Maria Maddalena Nicoletti, Giuseppe Argenziano

**Affiliations:** ^1^ Dermatology Unit University of Campania L. Vanvitelli Naples Italy

**Keywords:** dermoscopy, longitudinal monitoring, melanocytic lesions, melanoma, overdiagnosis

## Abstract

Dermoscopy has enabled the detection of increasingly subtle melanocytic lesions and has contributed to a marked rise in early melanoma diagnoses. However, this shift has not been matched by a proportional reduction in mortality, raising the question of whether all lesions currently classified as early melanoma are biologically meaningful. Part of the problem lies in the definition itself. “Early melanoma” is often treated as a uniform entity, yet it likely includes lesions with widely different biological trajectories. Dermoscopy, based on the recognition of morphological patterns, does not access this biological dimension. It captures structure, not behavior and therefore operates within an inherent limitation: atypia does not necessarily equate to clinically relevant malignancy. In practice, lesions may appear suspicious yet remain unchanged over time, while others evolve. This simple observation introduces a critical dimension that static assessment cannot capture. Stability challenges the significance of atypia, while change raises concern—but neither fully resolves the underlying uncertainty. Dermoscopy, therefore, does not diagnose early melanoma in a definitive sense. It identifies lesions that warrant attention within a biologically heterogeneous spectrum. Sequential observation does not eliminate ambiguity, but places it in context, where time becomes part of the evaluation rather than an afterthought. The key issue may not be how early melanoma can be detected, but how to distinguish lesions that will evolve from those that will not.

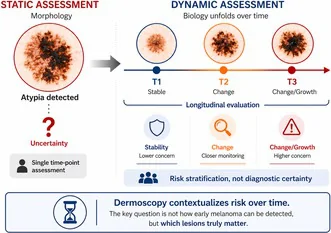

## Graphical abstract

Comparison between static and dynamic dermoscopic assessment of melanocytic lesions. Static evaluation relies on a single time‐point morphological assessment and may leave uncertainty regarding biological relevance. In contrast, longitudinal dermoscopic follow‐up incorporates temporal changes, allowing lesions to be stratified according to stability, change, or growth. This dynamic approach provides biological context for risk stratification and supports more informed clinical decision‐making, rather than relying solely on baseline morphology.

**Table:** 

Key Questions	Author Insights
Since dermoscopic pattern recognition is trained on histopathologically confirmed cases (which themselves are subject to interpretive ambiguity), does this circularity limit dermoscopy's diagnostic ceiling, or does it argue for recalibrating the reference standard?	This circularity does not simply limit dermoscopy—it reveals the limits of the reference standard itself. Dermoscopy cannot outperform histopathology if it is trained on it, particularly when histopathologic interpretation is variable in early and borderline lesions. Rather than a ceiling of dermoscopy, this reflects a shared uncertainty. It argues for recalibrating the idea of a single gold standard and moving toward an integrated, probabilistic model of diagnosis
Could sequential dermoscopy and change‐over‐time algorithms resolve the ambiguity that single‐encounter pattern recognition cannot, or does monitoring simply defer rather than resolve the same diagnostic uncertainty?	Sequential dermoscopy does not resolve uncertainty, but it reframes it. Time adds a critical dimension that static morphology cannot capture, highlighting biological behavior rather than appearance alone. However, stability does not exclude histopathologic melanoma, and change does not necessarily imply aggressive potential. Monitoring therefore does not eliminate ambiguity but contextualizes it—often more meaningfully than a single encounter

## 1 Introduction

Over the past decades, the widespread adoption of dermoscopy has profoundly transformed the early detection of melanoma, contributing to a significant increase in the diagnosis of thin lesions and improved patient outcomes [1, 2]. However, this success has been accompanied by a parallel and increasingly debated phenomenon: a marked rise in melanoma incidence without a proportional decrease in mortality [3]. This discrepancy has raised critical concerns regarding overdiagnosis and the biological relevance of lesions currently classified as melanoma, particularly at the earliest stages.

At the core of this debate lies a fundamental yet often overlooked question: what do we actually mean by “early melanoma”? While traditionally considered a precursor stage within a linear progression model from benign nevus to invasive malignancy, this concept has been increasingly challenged. In this context, the assumption that all lesions diagnosed as “early melanoma” represent clinically meaningful disease requiring intervention becomes far less certain.

Dermoscopy has been positioned as a key tool in the identification of melanoma at its earliest stages, based on the recognition of specific morphological patterns [4]. Yet, dermoscopic features are indirect expressions of underlying histopathological structures, which themselves may not reliably reflect the biological behavior of a given lesion. The implicit expectation that dermoscopy can diagnose early melanoma therefore rests on a series of assumptions: that early melanoma is a clearly defined entity, that its morphological correlates are consistently recognizable, and that these correlates correspond to clinically relevant malignancy. Each of these assumptions deserves critical scrutiny.

In this review, we aim to move beyond a purely descriptive appraisal of dermoscopic criteria and instead address a more fundamental question: can dermoscopy truly diagnose early melanoma, or does it rather identify patterns of risk within a biologically unstable and temporally dynamic process?

## 2 What Is “Early Melanoma”?

The concept of “early melanoma” is widely used in both clinical practice and research, yet its definition remains inherently uncertain. Traditionally, melanoma has been framed within a linear progression model, in which benign melanocytic nevi evolve through intermediate stages into invasive malignancy [5, 6]. Within this paradigm, “early melanoma” is implicitly understood as a biologically initial phase of this continuum, often equated with melanoma in situ or thin invasive melanoma. However, increasing evidence challenges the universality of this progression model and calls into question the biological coherence of the category itself.

Molecular and genomic studies have demonstrated that melanocytic neoplasms do not follow a single, uniform evolutionary pathway. Distinct subsets of melanoma appear to arise de novo, without a precursor nevus, while others may share genetic alterations with benign or so‐called “intermediate lesions” without necessarily progressing toward aggressive behavior [7]. This heterogeneity suggests that the term “early melanoma” may encompass lesions with markedly different biological potentials, ranging from indolent proliferations to truly aggressive neoplasms detected at an early stage.

From a histopathological perspective, the challenge is further compounded by the existence of morphological borderline and intermediate melanocytic tumors, often categorized as atypical intraepidermal proliferations, SAMPUS (superficial atypical melanocytic proliferations of uncertain significance), MELTUMP (melanocytic tumors of uncertain malignant potential), and Spitz melanocytoma [8, 9]. These entities expose the limitations of the conventional benign–malignant dichotomy and make clear that diagnostic uncertainty is not merely a clinical inconvenience but an intrinsic feature of melanocytic pathology. Even among expert dermatopathologists, substantial interobserver variability persists, especially in thin, in situ, and borderline lesions. If the histopathologic gold standard itself is unstable, the expectation that dermoscopy can confidently identify early melanoma becomes immediately more problematic [10].

The recent WHO classification of skin tumors has attempted to address this complexity by introducing a pathway‐based model of melanomagenesis, recognizing multiple distinct routes to melanoma rather than a single linear sequence [11, 12]. While this framework represents an important conceptual advance, its translation into daily diagnostic practice remains incomplete. In particular, it does not fully resolve the ambiguity surrounding lesions that fall at the interface between benignity and malignancy, where both biological behavior and clinical management remain uncertain [13].

In this context, the term “early melanoma” risks functioning more as a diagnostic label than as a biologically meaningful entity. Its use may inadvertently reinforce the assumption that all such lesions represent clinically significant disease requiring intervention, thereby contributing to overdiagnosis and potential overtreatment. A critical reappraisal of this concept is therefore essential, especially when evaluating the capabilities and limitations of diagnostic tools such as dermoscopy.

## 3 What Does Dermoscopy Actually See?

Through the identification of pigment networks, globules, streaks, regression structures, and vascular patterns, dermoscopy provides a detailed representation of lesion architecture that has been shown to improve diagnostic accuracy compared to naked‐eye examination alone [14]. However, the nature of this information remains fundamentally morphological, raising important questions about its relationship to the underlying biology of melanocytic tumors.

Dermoscopy does not evaluate cellular atypia, mutational status, metastatic potential, or biological aggressiveness. It captures optical correlates of tissue architecture. These are useful, but they remain indirect. More importantly, they represent static snapshots of a process that is dynamic, heterogeneous, and not necessarily linear. A lesion may look atypical at a given moment without ever acquiring clinically relevant malignant potential, while another may evolve rapidly despite initially subtle or bland features. Morphology alone does not exhaust biology.

This limitation becomes evident when one considers the imperfect correspondence between dermoscopic criteria and diagnosis. Several dermoscopic features traditionally associated with melanoma, such as irregular networks, atypical globules, or regression structures, may also be observed in benign lesions, including dysplastic nevi and Spitz nevi [15, 16]. Conversely, early melanomas—especially those arising de novo—may lack overtly atypical features and appear dermoscopically subtle or even indistinguishable from benign lesions [17]. This overlap underscores a fundamental limitation: dermoscopy operates within a probabilistic framework, in which features increase or decrease the likelihood of malignancy but do not establish it definitively.

Moreover, dermoscopic evaluation is inherently influenced by observer‐dependent factors, including experience, training, and pattern recognition strategies. Although standardized algorithms and checklists—such as the ABCD rule, the 7‐point checklist, and pattern analysis—have improved consistency, interobserver variability remains a significant issue, particularly in equivocal lesions [18]. Even among experts, disagreement is not uncommon when assessing equivocal melanocytic tumors, reflecting the intrinsic ambiguity of the features being evaluated.

The clinical consequences of these limitations are substantial. In practice, dermoscopy contributes significantly to select lesions that may satisfy histopathologic criteria for melanoma while remaining biologically uncertain. Dermoscopy therefore contributes not only to earlier detection, but also potentially to overdiagnosis, particularly when static atypia is interpreted as synonymous with meaningful disease [19, 20].

Importantly, dermoscopy has never been intended to distinguish biologically aggressive from indolent melanoma directly. Rather, dermoscopic criteria were developed to identify morphologic irregularities associated with an increased likelihood of melanoma and to assist clinicians in selecting lesions that warrant closer evaluation or excision. Nevertheless, in everyday clinical practice, atypical dermoscopic morphology is often implicitly interpreted as a surrogate for biological significance, particularly in the context of early and borderline lesions. This creates an important conceptual tension: while dermoscopy successfully identifies structural atypia, it cannot determine with certainty whether such atypia reflects clinically meaningful malignant potential. In this sense, the limitation does not lie in dermoscopy alone, but in the broader assumption that morphology‐based assessment, whether clinical, dermoscopic, or histopathologic, can fully capture the biological behavior of melanocytic tumors.

For these reasons, dermoscopy does not “see” melanoma in a direct or definitive sense. It identifies morphologic irregularities that may correlate with malignancy, but only imperfectly and only within a broader context of clinical interpretation. Its real function is not to diagnose early melanoma as a fixed entity, but to estimate risk within a continuum of morphologic abnormalities.

## 4 Concept of Change: Why Time Matters More Than Static Morphology

A further critical limitation of dermoscopy (as is the case for histopathology) lies in its fundamentally static nature. Dermoscopic evaluation is typically based on the assessment of morphological features at a single time point, yet melanocytic lesions are dynamic entities whose biological significance may only become apparent over time. This temporal dimension is often underappreciated, but it may be more informative than morphology alone. In clinical practice, it is not uncommon to encounter flat, clinically and dermoscopically atypical lesions that remain stable for years under digital monitoring. These lesions may appear “suspicious” according to established criteria, yet show no meaningful evolution, raising questions about the biological relevance of their atypia.

Paradoxically, when such lesions are eventually excised—often after prolonged stability—the histopathological diagnosis may be of melanoma in situ (Figures 1, 2, 3). This observation introduces a fundamental dilemma: if the lesion had remained morphologically unchanged over time, was it biologically malignant from the outset, or does the label of melanoma in situ encompass lesions with limited or negligible malignant potential? In this context, the concept of malignancy becomes temporally ambiguous, and the distinction between early melanoma and indolent atypical proliferation is blurred (Figure 4, adapted from Welch [21]).

**FIGURE 1:**
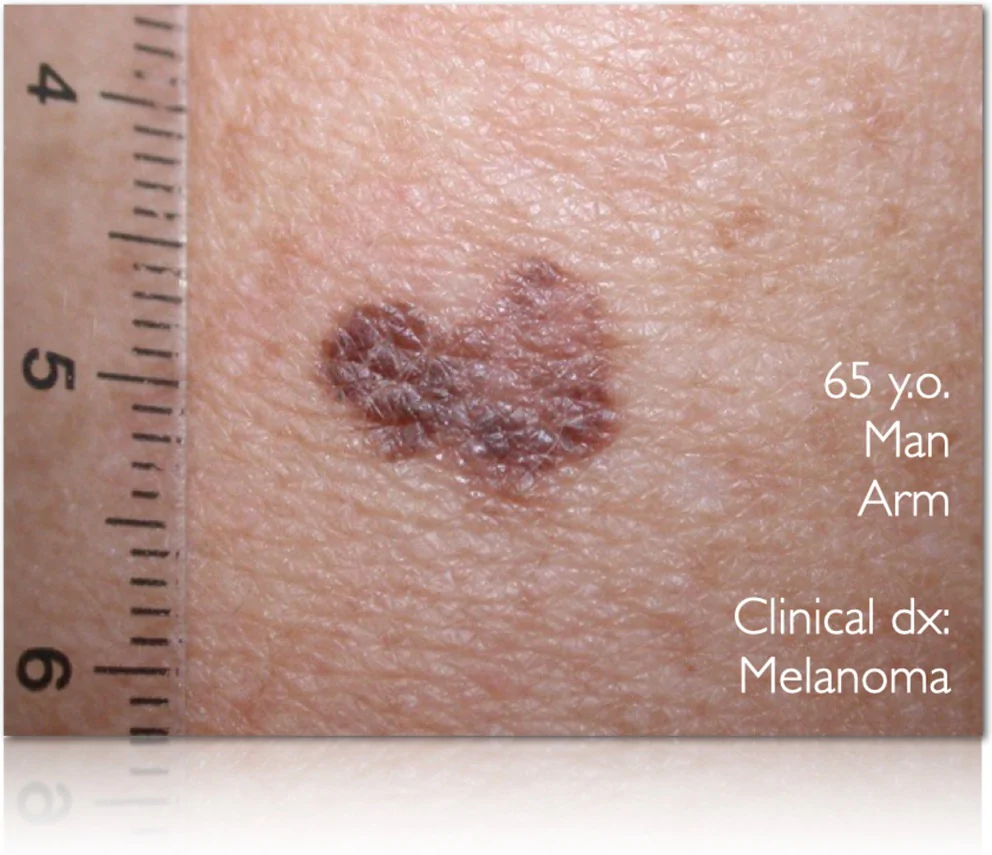
Clinically atypical melanocytic lesion at a single time point. A flat pigmented lesion showing asymmetry and irregular pigmentation on clinical examination.

**FIGURE 2:**
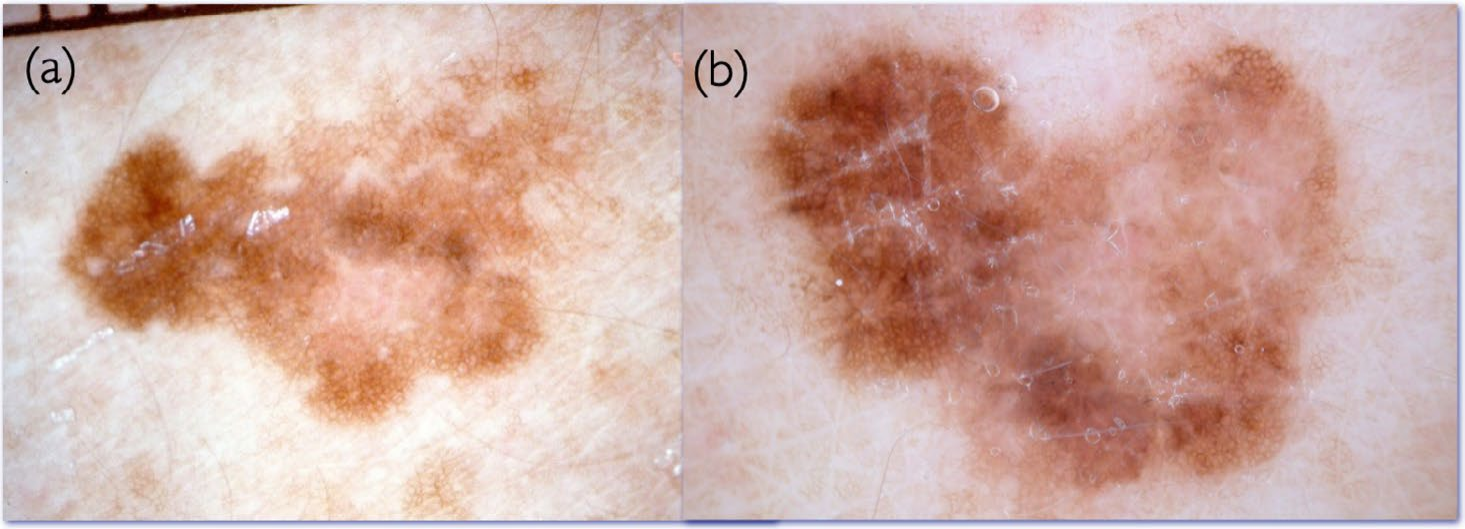
Long‐term dermoscopic monitoring of an atypical melanocytic lesion. Sequential dermoscopic images obtained over a 10‐year interval (a) –1993 and (b) –2003.

**FIGURE 3:**
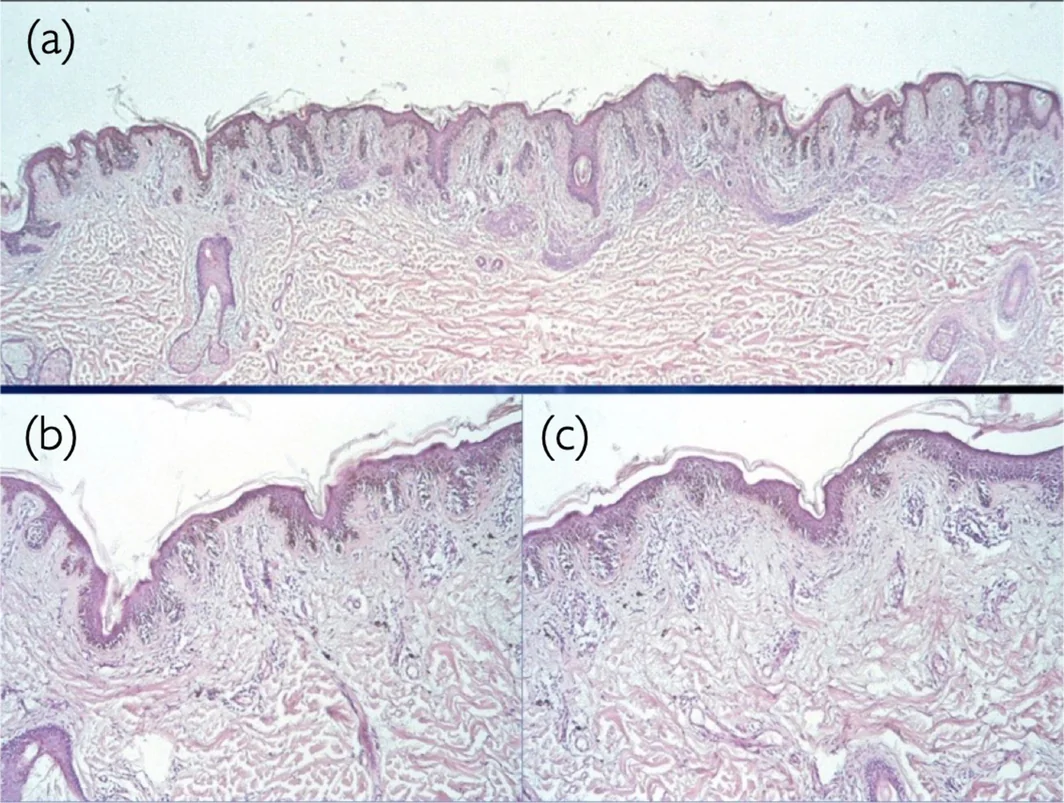
Histopathologic examination of the previous lesion monitored over time by long‐term sequential dermoscopy imaging revealing melanoma in situ with subtle architectural atypia. (Hematoxylin and eosin stain; low‐power view (a) and higher magnification (b and c)).

**FIGURE 4:**
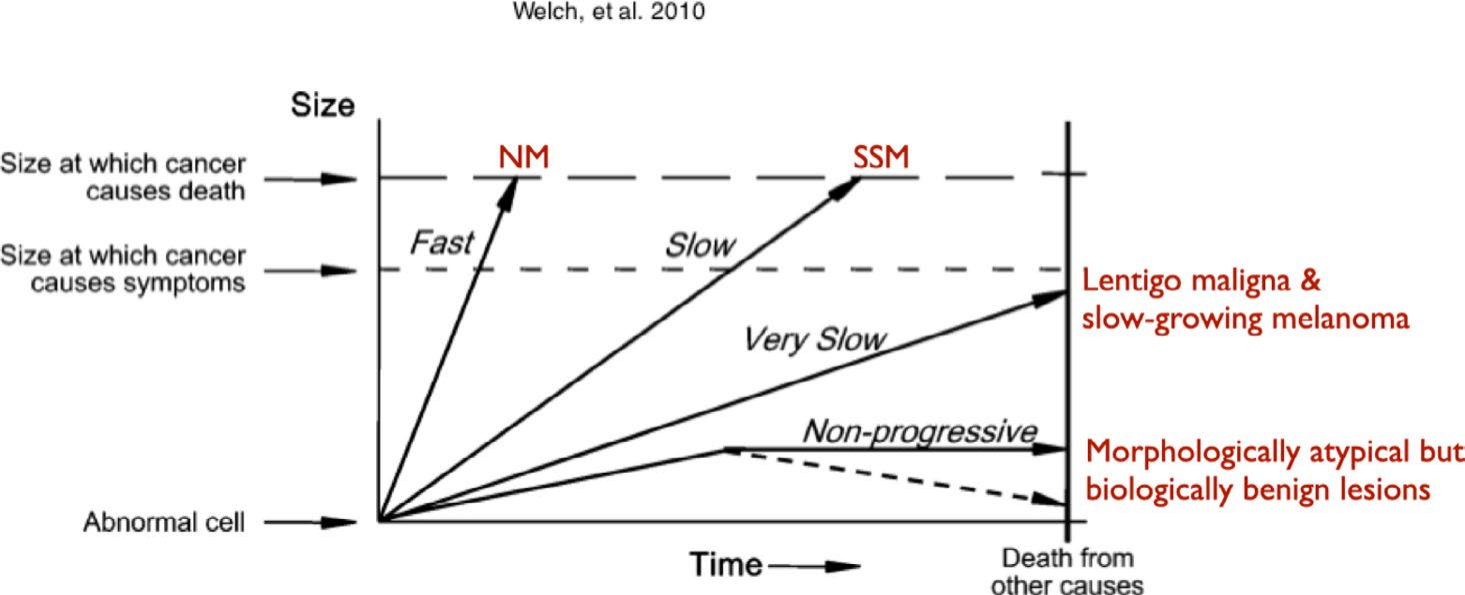
Schematic representation of variable tumor growth trajectories, ranging from rapidly progressive to indolent or non‐progressive lesions. While some tumors reach a size associated with symptoms or death, others remain clinically silent or never progress within a patient's lifetime. This model illustrates the biological basis of overdiagnosis and provides a conceptual framework for understanding the detection of early melanoma. (Adapted from Welch [21]).

These considerations highlight the importance of change over time as a critical dimension in the assessment of melanocytic lesions. While dermoscopic criteria focus on structural irregularities at a given moment, longitudinal evolution may provide more meaningful insight into biological behavior. A lesion that actively changes—developing new structures, asymmetry, or growth—may carry a different level of risk compared to one that remains morphologically stable despite atypical features. However, current diagnostic frameworks remain largely anchored to static morphology, potentially overemphasizing atypia while underestimating temporal stability.

In this light, the integration of sequential dermoscopic imaging and digital monitoring represents not only a practical tool but also a conceptual shift—from a purely morphological diagnosis to a time‐based assessment of risk [22]. In this context, change over time may be more informative than morphology at a single time point. Sequential digital dermoscopy should not be regarded merely as a practical follow‐up strategy for equivocal lesions, but as a conceptual shift in how risk is assessed. It allows clinicians to move beyond the assumption that atypia equals danger and to recognize that temporal behavior may provide a more meaningful clue to biological significance than static appearance alone.

Nevertheless, the practical implementation of sequential dermoscopic monitoring also deserves consideration. Longitudinal imaging requires dedicated equipment, digital storage systems, time, training, and structured follow‐up pathways, resources that may not be equally available across all clinical settings. In many parts of the world, access to serial digital dermoscopy remains largely confined to referral centres and high‐risk melanoma clinics. Importantly, the concept of change over time should not be interpreted as a recommendation for universal long‐term monitoring of all atypical lesions. Rather, sequential imaging may be most appropriately viewed as a selective risk‐stratification strategy for equivocal melanocytic lesions in which immediate excision or reassurance are both uncertain. Even simple baseline clinical or dermoscopic documentation, when available, may improve longitudinal assessment and contextual interpretation over time.

In a field where many lesions lie in a gray zone, time is not an accessory variable; it is part of the diagnosis. This perspective also helps explain why some lesions classified as melanoma in situ remain clinically silent for prolonged periods, while others show measurable evolution and demand intervention. It suggests that our current frameworks may overvalue static atypia while undervaluing stability. More broadly, it challenges the notion that malignancy can always be inferred from appearance alone. If a lesion is dynamic, time matters. If it is stable, time matters just as much.

## 5 The Limits of Early Detection

The paradigm of early detection has long been central to melanoma management, grounded in the assumption that diagnosing lesions at an earlier stage necessarily translates into improved survival. While this principle holds true for many aggressive malignancies, its application to melanoma has become increasingly complex and, in some respects, controversial. Epidemiological data from several countries have demonstrated a substantial rise in melanoma incidence over recent decades, particularly in thin and in situ lesions, without a corresponding proportional decline in mortality [23, 24]. This divergence has raised the possibility that a significant proportion of lesions currently diagnosed as melanoma may represent biologically indolent tumors that would not have progressed to cause harm if left undetected (Figure 4).

Dermoscopy has undoubtedly contributed to biopsy more and more lesions that may satisfy histopathological criteria for melanoma. In such cases, the decision to excise is often driven by the presence of atypical dermoscopic features rather than clear evidence of aggressive biological potential. As a result, dermoscopy may facilitate the detection of lesions that meet histopathological criteria for melanoma, but whose clinical relevance remains uncertain [25].

This phenomenon is closely aligned with the concept of overdiagnosis, defined as the detection of a disease that would not have become clinically significant within a patient's lifetime. In melanoma, overdiagnosis is particularly challenging to quantify, given the lack of reliable markers distinguishing indolent from aggressive lesions at the time of diagnosis. Nevertheless, indirect indicators—including the disproportionate increase in early‐stage diagnoses, stable or only modestly declining mortality rates, and substantial interobserver variability in histopathological interpretation—support the notion that overdiagnosis is a relevant and underrecognized issue [26, 27].

Importantly, the implications of overdiagnosis extend beyond epidemiological considerations. The diagnosis of melanoma, even at an early stage, carries significant psychological, clinical, and economic consequences. Patients may undergo repeated surgical procedures, long‐term follow‐up, and experience anxiety related to a cancer diagnosis, even when the biological behavior of the lesion is uncertain. In this context, the role of diagnostic tools should be critically examined not only in terms of their ability to detect disease, but also in relation to the downstream effects of that detection.

## 6 Beyond Dermoscopy: Toward an Integrated and Probabilistic Model of Diagnosis

The limitations of dermoscopy in diagnosing early melanoma should not be interpreted as a failure of the technique itself, but rather as an indication that the problem it attempts to solve is inherently more complex than originally assumed. If melanoma—particularly at its earliest stages—is not a clearly bounded biological entity, then no single diagnostic modality, regardless of its resolution or sophistication, can be expected to identify it with certainty.

Emerging technologies such as reflectance confocal microscopy (RCM), optical coherence tomography (OCT), and line‐field confocal OCT (LC‐OCT) have significantly expanded our ability to visualize skin structures at near‐histological resolution in vivo [28, 29]. These tools offer a more direct assessment of cellular and architectural features, potentially bridging part of the gap between clinical observation and histopathological interpretation. At the same time, advances in artificial intelligence and machine learning promise to enhance pattern recognition beyond human capabilities, while molecular and genomic profiling aims to redefine melanocytic tumors based on their biological signatures rather than their morphology [30].

Yet, despite these advances, the expectation that any of these technologies will “solve” the problem of early melanoma diagnosis may be fundamentally misplaced. Each modality, including dermoscopy, captures a different layer of a multidimensional process: morphology, cellular architecture, molecular alterations, or statistical pattern recognition. None of these layers alone fully defines malignancy, particularly in lesions that exist at the interface between benign and malignant morphology.

Rather than seeking a definitive diagnostic tool, a more realistic and conceptually sound approach may be to embrace a probabilistic and integrative model of diagnosis. In such a model, dermoscopy is not replaced but repositioned as an initial, highly sensitive screening tool that identifies lesions requiring further characterization. Sequential dermoscopy imaging and advanced imaging techniques refine this assessment by providing additional dynamic, structural, and cellular information, while molecular and computational approaches contribute complementary insights into biological potential. Diagnosis, in this framework, is no longer a binary act but a dynamic process of risk stratification across multiple dimensions.

## 7 Conclusion

The question of whether dermoscopy can allow diagnosing early melanoma ultimately exposes a deeper conceptual limitation: the assumption that early melanoma is a stable, identifiable entity that can be recognized based on morphology alone. As discussed, both the definition of melanoma at its earliest stages and the tools used to detect it are affected by significant biological and interpretative uncertainty.

Dermoscopy has undeniably improved the differentiation of benign and malignant skin lesions and remains an essential component of modern dermatologic practice. However, it does not allow diagnosing melanoma in a definitive sense; rather, it identifies morphological patterns associated with an increased probability of malignancy within an imperfect and evolving classification system. Nowhere is this limitation more evident than in borderline and stable atypical lesions, where suspicious features may persist unchanged over time, challenging the assumption that morphological atypia necessarily reflects biologically meaningful disease.

The concept of change over time emerges as a critical, yet underappreciated, dimension in this context. A lesion that evolves carries a different biological implication from one that remains stable, even when both display atypical features. By focusing predominantly on static morphology, current diagnostic frameworks risk overestimating the significance of atypia while underrecognizing the importance of temporal behavior.

In this perspective, dermoscopy should not be viewed as a tool that allows diagnosing early melanoma, but as one that contributes to a broader process of risk assessment, ideally integrated with longitudinal observation and advanced imaging. Accepting the limits of morphological diagnosis does not weaken clinical practice; rather, it allows for a more nuanced and biologically grounded approach to melanocytic lesions.

Ultimately, the challenge is not to improve our ability to label lesions as melanoma at increasingly earlier stages, but to better understand which lesions truly matter.

## Funding

The authors have nothing to report.

## Conflicts of Interest

The authors declare no conflicts of interest.

## Data Availability

The data that support the findings of this study are available from the corresponding author upon reasonable request.
